# Supplementary material for: Experience-dependent flexibility in a molecularly diverse central-to-peripheral auditory feedback system
Source: eLife. 2023 Mar 6;12:e83855. doi: 10.7554/eLife.83855 (PMC10147377; doi:10.7554/eLife.83855)
Supplement: Supplementary file 3. — Results of single linear regression and correlation analysis between Calca-GFP and the indicated measures. [file elife-83855-supp3.docx]

| **Measure** | **Pearson's *r*** | ***p*** | ***R^2^*** | ***n* (neurons)** | **Mean** | **SD** |
| --- | --- | --- | --- | --- | --- | --- |
| Membrane capacitance | -0.15 | 0.42 | 0.22 | 31 | 19.36 pF | 3.18 pF |
| Input resistance | 0.081 | 0.60 | 6.60E-03 | 45 | 324.6 MΩ | 186.02 MΩ |
| Outward K+ current magnitude | 0.1 | 0.53 | 0.01 | 43 | 2.78 nA | 1.62 nA |
| Steady-state inward current | -0.21 | 0.17 | 0.047 | 42 | -183.18 pA | 121.29 pA |
| A-type K+ current amplitude | -0.16 | 0.54 | 0.026 | 17 | 412.63 pA | 285.89 pA |
| Fast component of time constant of decay of fast-inactivating K+ current | -0.18 | 0.5 | 0.031 | 17 | 7.9 ms | 6.86 ms |
| Spontaneous firing rate | -0.28 | 0.35 | 0.08 | 13 | 8.6 Hz | 5.9 Hz |
| Rheobase | 0.06 | 0.76 | 0.004 | 24 | 13.3 pA | 8.20 pA |
| Action potential threshold | -0.25 | 0.26 | 0.06 | 24 | -49.08 mV | 6.12 mV |
| Amplitude from baseline of first spike evoked at rheobase | 0.01 | 0.95 | 0.0016 | 24 | 69.04 mV | 16.75 mV |
| Number of action potentials evoked by a 10 pA current injection | -0.08 | 0.75 | 0.006 | 24 | 4.5 | 3.11 |
| Input-output curve slope | -0.33 | 0.11 | 0.16 | 19 | 0.2 | 0.14 |

**Table S3 (Related to Figure 6). Physiological properties of LOCs do not correlate with CGRP-GFP fluorescence intensity.** Results of single linear regression and correlation analysis between CGRP-GFP and the indicated measures.
