# Supplementary material for: Experience-dependent flexibility in a molecularly diverse central-to-peripheral auditory feedback system
Source: eLife. 2023 Mar 6;12:e83855. doi: 10.7554/eLife.83855 (PMC10147377; doi:10.7554/eLife.83855)
Supplement: Supplementary file 4. — Adjusted p-values from Kruskal-Wallis multiple-comparisons test for the indicated cell types and ages. Data is shown in Figure 7A–D. [file elife-83855-supp4.docx]

| **Comparison** | **Calca** | **Calcb** | **Ucn** | **NPY** |
| --- | --- | --- | --- | --- |
| LOC P1 vs. LOC P5 | <0.0001 | 0.1637 | 0.0728 | >0.9999 |
| LOC P1 vs. LOC P28 | <0.0001 | <0.0001 | <0.0001 | <0.0001 |
| LOC P1 vs. MOC P1 | >0.9999 | >0.9999 | >0.9999 | 0.5646 |
| LOC P1 vs. MOC P5 | 0.0611 | 0.6236 | >0.9999 | >0.9999 |
| LOC P1 vs. MOC P28 | <0.0001 | 0.0991 | >0.9999 | >0.9999 |
| LOC P5 vs. LOC P28 | <0.0001 | 0.0202 | <0.0001 | <0.0001 |
| LOC P5 vs. MOC P1 | <0.0001 | 0.0032 | 0.1595 | >0.9999 |
| LOC P5 vs. MOC P5 | 0.0979 | >0.9999 | 0.7234 | >0.9999 |
| LOC P5 vs. MOC P28 | 0.0755 | >0.9999 | 0.158 | >0.9999 |
| LOC P28 vs. MOC P1 | <0.0001 | <0.0001 | <0.0001 | <0.0001 |
| LOC P28 vs. MOC P5 | <0.0001 | 0.1575 | <0.0001 | <0.0001 |
| LOC P28 vs. MOC P28 | <0.0001 | 0.3052 | <0.0001 | <0.0001 |
| MOC P1 vs. MOC P5 | 0.1369 | 0.0332 | >0.9999 | >0.9999 |
| MOC P1 vs. MOC P28 | <0.0001 | 0.0025 | >0.9999 | >0.9999 |
| MOC P5 vs. MOC P28 | <0.0001 | >0.9999 | >0.9999 | >0.9999 |

**Table S4 (Related to Figure 7). Results of statistical tests comparing peptide expression between OCN groups at different ages.** Adjusted p-values from Kruskal-Wallis multiple-comparisons test for the indicated cell types and ages. Data is shown in Figure 7A-D.
